# Supplementary material for: Comprehensive Transcriptomic Analysis of Heterotrophic Nitrifying Bacterium Klebsiella sp. TN-10 in Response to Nitrogen Stress
Source: Microorganisms. 2022 Feb 3;10(2):353. doi: 10.3390/microorganisms10020353 (PMC8876665; doi:10.3390/microorganisms10020353)
Supplement: Supplementary file 1 [file microorganisms-10-00353-s001.zip › microorganisms-1529594-supplementary.pdf]

Table S1 Genome statistics of strain TN-10

Feature	Value
Genome size (bp)	6036048
GC content %	55.67
DNA coding (bp)	5289228
Gene average length (bp)	907.24
Total genes	5830
GC content in Gene Region (%)	56.97
Gene/Genome (%)	87.62
tRNA numbers	84
rRNA numbers	45

Table S2 Statistics of each sample sequencing data

Sample name	Raw reads	Clean reads	Clean error rate (%)	Clean Q20(%)	Clean Q30(%)	CDS mapped reads	CDS mapped reads
group-L (a)	22693616	22026494	0.0119	98.62	95.76	17357005	78.8
group-L (b)	19605776	19170898	0.0123	98.41	95.16	11892665	62.03
group-L (c)	22581680	22117264	0.0123	98.42	95.18	13652650	61.73
group-M (a)	20018582	19544294	0.0123	98.41	95.16	11339677	58.02
group-M (b)	20258310	19828272	0.0123	98.43	95.20	12959144	65.36
group-M (c)	21651766	21114688	0.0120	98.57	95.57	11932557	56.51
group-H (a)	22559636	21966912	0.0118	98.64	95.77	13481484	61.37
group-H (b)	22050566	21425880	0.0120	98.54	95.50	14026585	65.47
group-H (c)	20397914	19933288	0.0121	98.50	95.37	13312697	66.79

Table S3 Primers used in this study

Genes (gene ID)	Primer	Oligonucleotide sequence (5'-3')
narK (Gene 2311)	Forward	TCTCCGTCCCGTGTCTTT
	Reverse	ACAACTGGTCGGTGGTAAACT
narG (Gene 2312)	Forward	AGCGAAAGTTCAGCACAGC
	Reverse	CAGGACGAACGCACGAAA
narI (Gene 2315)	Forward	TATGGACGGTAGCGAAATGA
	Reverse	AACGGGAACAGCACAAACA
gdhA (Gene 3066)	Forward	ACCACCTACCTGCGTCAAA
	Reverse	CGGATAGTACCGTCGTCCA
sdhB (Gene 3958)	Forward	ACCCTGGAAGCGGAAGA
	Reverse	TCGGAGCCACAAACCC
16S rRNA	Forward	ATCCTGGCTCAGATTGAACGC
	Reverse	CGGGCAGTTTCCCAGACATTAC

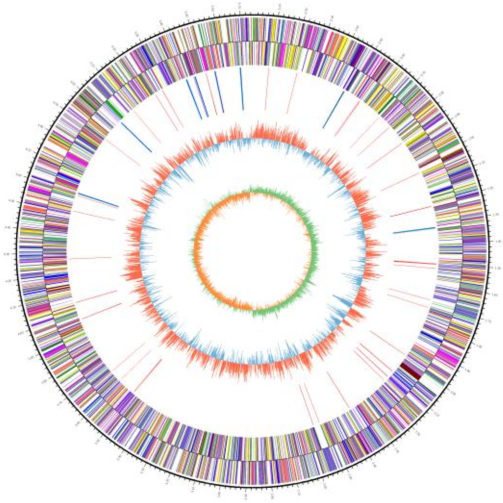

Figure S1. Circular genome map of *Klebsiella* sp. TN-10. From the inside to outside: GC-skew; GC content; rRNA and tRNA; CDS (Coding sequence) on the reverse strand; CDS (Coding sequence) on the forward strand; the whole chromosome.

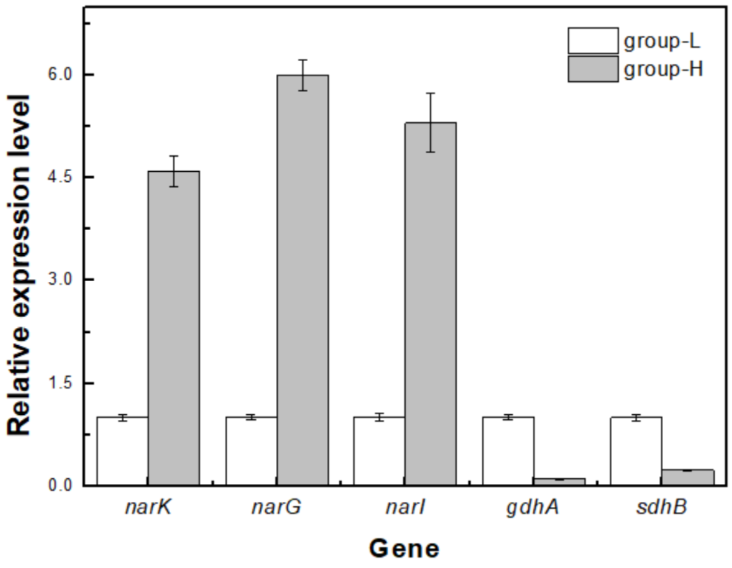

Figure S2. Gene transcriptional expression by q-PCR analysis in *Klebsiella* sp. TN-10 under different nitrogen stresses.
